# Vitamin D Alleviates Cadmium-Induced Inhibition of Chicken Bone Marrow Stromal Cells’ Osteogenic Differentiation In Vitro

**DOI:** 10.3390/ani13152544

**Published:** 2023-08-07

**Authors:** Xishuai Tong, Ying Zhang, Yutian Zhao, Yawen Li, Tan Li, Hui Zou, Yan Yuan, Jianchun Bian, Zongping Liu, Jianhong Gu

**Affiliations:** 1 Joint International Research Laboratory of Agriculture and Agri-Product Safety of the Ministry of Education of China, Institutes of Agricultural Science and Technology Development, College of Veterinary Medicine, Yangzhou University, Yangzhou 225009, China; xstong@yzu.edu.cn (X.T.); zouhui@yzu.edu.cn (H.Z.); yuanyan@yzu.edu.cn (Y.Y.); jcbian@yzu.edu.cn (J.B.); 2 Jiangsu Co-Innovation Center for Prevention and Control of Important Animal Infectious Diseases and Zoonoses, Yangzhou 225009, China; 3 Jiangsu Key Laboratory of Zoonosis, Yangzhou 225009, China; 4 National Health Commission Key Laboratory of Parasitic Disease Control and Prevention, Jiangsu Provincial Key Laboratory on Parasite and Vector Control Technology, Jiangsu Institute of Parasitic Diseases, Wuxi 214064, China; zhangying0707@yeah.net

**Keywords:** lα, 25-(OH)_2_D_3_, cadmium (Cd), bone marrow stromal cells (BMSCs), osteogenic differentiation, White Leghorn

## Abstract

**Simple Summary:**

The most physiologically active form of vitamin D is lα, 25-dehydroxyvitamin D_3_ (lα, 25-[OH]_2_D_3_), which participates in the bone metabolic homeostasis of animals. In addition to the bone matrix, bone also includes bone-related cells, such as bone marrow stromal cells (BMSCs), osteoblasts, osteoclasts, etc. BMSCs can differentiate into osteoblasts, which are regulated by the Runt-related transcription factor 2 (Runx2) and other factors. Furthermore, type Ⅰ collagen (Col1) is synthesized by osteoblasts and combines with hydroxyapatite to form normal bone matrix. lα, 25-(OH)_2_D_3_ promotes the secretion of Col1 in animals and stimulates bone mineralization. In addition, environmental heavy metal has accumulated toxicity in the different organs and tissues of animals, including in bone. Cadmium (Cd) is an element of heavy metals in nature that has osteotoxicity, including the destruction of the bone matrix and damage of bone-related cells (such as BMSCs and osteoblasts), and even inducing the apoptosis of bone-related cells. In this study, despite Cd having a certain toxicity on BMSCs and osteogenic differentiation from chicken embryos, our assessment of Cd osteotoxicity via lα, 25-(OH)_2_D_3_ suggested this mitigating effect was, indeed, improved. These signs indicate that lα, 25-(OH)_2_D_3_ can alleviate Cd-induced osteogenic toxicity in White Leghorn chickens in vitro.

**Abstract:**

Vitamin D is a lipid soluble vitamin that is mostly used to treat bone metabolism-related diseases. In this study, the effect of Cd toxicity in vitro on osteogenic differentiation derived from BMSCs and the alleviating effect of lα, 25-(OH)_2_D_3_ were investigated. Cell index in real time was monitored using a Real-time cell analyzer (RTCA) system. The activity of alkaline phosphatase (ALP), and the calcified nodules and the distribution of Runx2 protein were detected using ALP staining, alizarin red staining, and immunofluorescence, respectively. Furthermore, the mitochondrial membrane potential and the apoptotic rate of BMSCs, the mRNA levels of *RUNX2* and type Ⅰ collagen alpha2 (*COL1A2*) genes, and the protein expression of Col1 and Runx2 were detected using flow cytometry, qRT-PCR and western blot, respectively. The proliferation of BMSCs and osteogenic differentiation were enhanced after treatment with different concentrations of lα, 25-(OH)_2_D_3_ compared with the control group. However, 5 μmol/L Cd inhibited the proliferation of BMSCs. In addition, 10 nmol/L lα,25-(OH)_2_D_3_ attenuated the toxicity and the apoptosis of BMSCs treated by Cd, and also promoted the osteogenic differentiation including the activity of ALP, and the protein expression of Col1 and Runx2. lα, 25-(OH)_2_D_3_ can alleviate cadmium-induced osteogenic toxicity in White Leghorn chickens in vitro.

## 1. Introduction

Vitamin D is a lipid soluble vitamin, and is mostly used to treat bone metabolism-related diseases in animals, such as Osteoporosis (OP), Osteomalacia (OM), Multiple Osteosclerosis (MS), and Rheumatoid Arthritis (RA) [1,2,3,4]. Previous studies have shown that dietary supplementation with vitamin D_3_ and calcium (Ca) has been observed to decrease the severity and the incidence of Tibial Dyschondroplasia (TD) in young broilers [5,6,7]. 7-dehydrocholesterol (7-DHC) derived from the skin of animal is converted to pro-vitamin D_3_ under ultraviolet (290–315 nm) irradiation [8]. Under the action of 25-hydroxylase in liver and 25-hydroxyvitamin D-1 alpha-hydroxylase (also known as Cyp27b1) in kidney, vitamin D_3_ generates lα, 25-dehydroxyvitamin D_3_ (lα, 25-[OH]_2_D_3_), which is the most physiologically active form and is involved in the dynamic homeostasis of Ca ions (Ca^2+^) in vivo [9]. Vitamin D stimulates the vitamin D receptor (VDR) to promote the absorption of Ca^2+^, normalizing the Ca^2+^ level of blood [10]. Active vitamin D accelerates the accumulation of Ca^2+^ into bones when serum Ca^2+^ is sufficient [11,12]. Osteoblast maturation derived from bone marrow stromal cells (BMSCs) is regulated by various transcription factors, such as Runt-related transcription factor 2 (Runx2) and type Ⅰ collagen (Col1) [13]. Runx2 is a pivotal regulatory factor during the differentiation of BMSCs into osteoblasts, which then activates the transcription of Col1 to achieve bone remodeling [14,15,16]. Thus, Runx2 and Col1 are the multifunctional regulatory factors that are essential for osteoblastic differentiation.

A previous study found that 24R, 25-(OH)_2_D_3_ (a metabolite of vitamin D_3_ prohormone 25-[OH]D_3_) can inhibit the proliferation of human bone marrow stromal cells (hBMSCs), while increasing alkaline phosphatase (ALP) activity and bone calcification [17]. Likewise, 10 nmol/L lα, 25-(OH)_2_D_3_ promoting osteoblast differentiation from hBMSCs by increasing the activity and the mRNA level of ALP, osteocalcin (OCN) production and Col1 expression [18]. Another study has demonstrated that lα, 25-(OH)_2_D_3_ enhanced the osteogenic differentiation of human periodontal ligament stem cells (hPDLSCs) [19]. However, Han et al. [20] reported that lα, 25-(OH)_2_D_3_ inhibited osteogenic differentiation from BMSCs by activating Wnt/β-catenin signaling and decreasing the expression of bone morphogenetic protein-2 (BMP-2). A high-dose concentration (100 nmol/L) of lα, 25-(OH)_2_D_3_ inhibited osteoblastic mineralization by suppressing the nuclear factor-κB (NF-κB) activation and Smad activation in primary osteoblast precursors or MC3T3 pre-osteoblastic cells [21]. In addition, lα, 25-(OH)_2_D_3_ plays a protective effect against hydrogen peroxide (H_2_O_2_)-induced apoptosis of nucleus pulposus-derived mesenchymal stem cells (NPMSCs) including the apoptosis rate and apoptosis-related protein expression, such as B cell lymphoma-2 (Bcl-2),Bcl-2 associated X-protein (Bax), and cleaved caspase-3 [22]. Therefore, vitamin D and/or its derivatives plays a vital role in the differentiation of osteoblast and/or BMSCs.

Cadmium (Cd) is a natural element that accumulates in various organs of animals and has the characteristics of chemical toxicity, long half-life and refractory decomposition [23,24,25]. Cd accumulates in the animal body through the food chain, and especially for the kidneys and bone is more toxic [26,27]. Importantly, Cd has a direct toxic effect on the tissues or cells of bone, and also indirectly affects bone metabolism through the toxicity of liver and kidney, including interfering with Ca and vitamin D metabolism [23,24,28,29,30]. Cd inhibits osteoblast differentiation derived from BMSCs, promotes adipocyte differentiation to inhibit BMSCs activity and bone matrix mineralization, thereby impairing bone metabolism and bone remodeling [29]. Likewise, Cd promoted the cytotoxicity of MC3T3-E1 subclone 14 cells through exportin-1 accumulation, DNA damage, the induction of cleaved caspase-3 protein, and inhibition of osteoblast-related protein expression, such as Runx2 and Col1 [31]. In addition, Cd induced apoptosis of primary osteoblasts in rats by upregulating the Bax/Bcl-2 ratio, and suppressing this action performed by autophagy activator rapamycin, while potentiated by autophagy inhibitor chloroquine (CQ) or small interfering RNA against Beclin1, which is an autophagy regulatory initiator [32]. In this study, the aim was to evaluate the alleviated effect of lα, 25-(OH)_2_D_3_ on Cd-inhibited osteogenic differentiation and Cd-enhanced cell apoptosis of BMSCs.

## 2. Materials and Methods

### 2.1. SPF- Grade Eggs

SPF-grade White Leghorn eggs were purchased from Shandong Yijida Biotechnology Co., Ltd. (Jining, China). The eggs were incubated at 37 °C and 60% humidity in the egg incubator (Dezhou Dingfeng Machinery Equipment Co., Ltd.; Dezhou, China) until they were 18 days old. This study strictly followed the recommendations of the *Guidelines for the Care and Use of Laboratory Animals* issued by the National Research Council. At the end of the incubation on day 18, all chicken embryos were humanely euthanized, and femur and tibia were separated for further testing. The experimental protocols were approved by the Animal Care and Use Committee of Yangzhou University (Approval ID: SYXK [Su] 2016–0020; Yangzhou, China).

### 2.2. Reagents

Alpha-minimum essential medium (α-MEM) and Dulbecco modified Eagle’s medium (DMEM) were purchased from Gibco (Grand Island, New York, NY, USA). Fetal bovine serum (FBS) was purchased from Beijing EallBio Biomedical Technology Co., Ltd. (Beijing, China). β-Glycerophosphate, BCIP^®^/NBT substrate solution, cadmium acetate, and Trizol reagent were purchased from Sigma–Aldrich (St. Louis, MO, USA). ALP staining and alizarin red staining kit were purchased from Beijing Solarbio Science & Technology Co., Ltd. (Beijing, China). 0.25% trypsin solution and electrochemiluminescence (ECL) chemiluminescence kit were purchased from Suzhou New Cell & Molecular Biotech Co., Ltd. (Suzhou, China). JC-1 staining solution was purchased from ThermoFisher (Waltham, MA, USA). Hoechst 33258 staining, BCA protein assay kit, RIPA lysis buffer and actin-tracker red-Rhodamine (phalloidin) probe were purchased from Beyotime Biotech. Inc. (Shanghai, China). HiScript Q RT SuperMix for qPCR (+gDNA wiper) was purchased from Nanjing Vazyme Biotech Co., Ltd. (Nanjing, Jiangsu, China). Hieff^®^ qPCR SYBR Green Master Mix was purchased from Yeasen Biotechnology (Shanghai) Co., Ltd. (Shanghai, China). 5 × SDS-PAGE Loading Buffer was purchased from Bio-Rad Laboratories, Inc. (Hercules, CA, USA). Polyclonal antibodies against Runx2, Col1, Bax and Bcl-2, and monoclonal antibody against cleaved caspase-3 were purchased from Beijing Bioss Biotechnology Co., Ltd. (Beijing, China). Polyclonal antibody against glyceraldehyde-3-phosphate dehydrogenase (GAPDH), horseradish peroxidase (HRP)-conjugated goat anti-mouse and HRP-conjugated goat anti-rabbit were purchased from Cell Signaling Technology, Inc. (Danvers, MA, USA).

### 2.3. Cell Culture and Treatment

The femur and tibia of 18-day-old chicken embryos were separated as previously reported [33]. The BMSCs were flushed using α-MEM without FBS. Cells were filtered using 300 mesh filter screens, and then centrifuged at 1000 r/min for 5 min. Cells (3 × 10^6^/mL at density) were resuspended in α-MEM supplement with 10% FBS at 5% CO_2_ with 37 °C. The medium was replaced every 2 days. The second generation of BMSCs was renewed to induced medium for osteogenic differentiation. Cells were divided into four group that included: only cells as the control group (only cells), 10 nmol/L lα,25-(OH)_2_D_3_ (pre-treated for 6 h) group, an only 5 μmol/L Cd group, and 10 nmol/L lα,25-(OH)_2_D_3_ + 5 μmol/L Cd group. 

### 2.4. Real-Time Cell Analyzer (RTCA) Dynamic Monitoring of BMSCs

DMEM medium (without FBS) was added to each E-plate well as reference lines for normalization using an xCelligence system (RTCA software 2.1; Roche, Basel, Switzerland) consistent with the manufacturer’s protocol as previously reported [34,35]. We placed the E-plate (a cell placement plate) in the 37 °C incubator for 30 min, and then cells (the density of cell at 5 × 10^3^/200 μL) were added to each well as previously described [34]. Next, the cells were treated with lα,25-(OH)_2_D_3_ in different concentrations (1, 10, and 100 nmol/L). The medium was replaced every 2 days. The normalized cell index (NCI) was calculated. Impedance was recorded every 15 min during the cell exponential proliferative phase [34].

### 2.5. ALP Staining and Alizarin Red Staining

The second-generation of BMSCs from chickens were seeded into a 6-well plate (the density of cells was 3 × 10^5^/mL), and changed to osteogenic induction medium (containing 10% FBS, 10 nmol/L dexamethasone, 10 mmol/L sodium β-glycerophosphate, and 50 μg/mL ascorbic acid) when they grew to about 75%. BMSCs was treated with induction medium for 7 days, and then the medium was discarded and washed with phosphate-buffered saline solution (PBS) 3–5 times. The cells were fixed in 4% paraformaldehyde solution for 30 min. Cell fluid was discarded, and then washed with PBS 3–5 times. BCIP^®^/NBT substrate solution was added to the cells for 30 min at room temperature (25 °C). The solution was discarded, and then washed with PBS twice. The picture was captured using a DMI 3000B inverted phase contrast microscope (Leica, Germany) for further observation.

In addition, the BMSCs were suspended in osteogenic induction medium for 21 days. The induction medium was discarded, and the cells were washed with PBS 3–5 times. The ethanol (at a percent of 90) was added with the cells for 30 min at room temperature (25 °C). The solution was discarded, and then washed with PBS 3–5 times. Alizarin red staining solution was added with the cells at room temperature (25 °C). The images were recorded using a DMI 3000B inverted phase contrast microscope (Leica, Germany).

### 2.6. Flow Cytometry Assay

Cells were collected and washed with PBS 2 times. Next, the cells were resuspended in 1 × Binding Buffer, and centrifuged at 1000 r/min for 10 min. The supernatant solution was discarded. Cells were incubated with Annexin V-FITC probe for 10 min, and then propidium iodide (PI) was added for 5 min at room temperature (25 °C) in the dark. PBS was added into each tube for detection of cell apoptosis using a FACS LSRFortess Flow cytometry assay machine (BD Biosciences; San Jose, CA, USA).

In addition, cells were digested using the 0.25% trypsin solution with EDTA for 30 s. The cells were collected using the 15 mL centrifuge tubes for washing with PBS twice. Cells were incubated with α-MEM (supplemented with 10% FBS). The supernatant solution was discarded. Then, the JC-1 staining solution, at a ratio of 1:1, was added for 20 min after the end of incubation. Finally, the cells were washed using the JC-1 staining solution twice for detection of cell mitochondrial membrane potential using a FACS LSRFortess Flow cytometry assay machine.

### 2.7. qRT-PCR

Total RNA was extracted using a Trizol reagent as previously reported [36]. Pure RNA was used to reverse transcribe into cDNA using a HiScript Q RT SuperMix kit for qPCR (+gDNA wiper). Then, the primer sequences (Table 1) were synthesized for qRT-PCR by using a Hieff^®^ qPCR SYBR Green Master Mix kit. The reaction methods were as follows: 95 °C for 30 s; 40 cycles of 95 °C for 5 s, 60 °C for 34 s; and 60 °C for 15 s. The mRNA expression of target genes was determined using the 2^−ΔΔCt^ method. Accordingly, *GAPDH* was used as the housekeeping gene for normalization.

### 2.8. Immunoblotting

Total protein was extracted using a lysis buffer as previously reported [33]. Equal amounts of denatured protein were divided into each tube. For immunoblotting, total proteins (30 μg) were separated using 12% or 10% sodium dodecyl sulfate-polyacrylamide gel electrophoresis (SDS-PAGE) gels. At the end of electrophoresis, the gels were transferred onto the polyvinylidene fluoride (PVDF) membranes with diameters of 0.22 μm (or 0.45 μm). At the end of protein transfer, the membranes were blocked with 5% free-fat milk buffer (the free-fat milk powder diluted into Tris-buffered saline solution with 0.1% Tween-20 [TBST]) for 1 h at room temperature (25 °C). Primary antibodies were used to dilute into different concentrations (Runx2, Col1, Bax, Bcl-2, cleaved caspase-3, and GAPDH at 1:1000), and then incubated at a low temperature (4 °C) for 12 h. All membranes were washed with TBST solution 5 times (5 min each time). Then, the membranes were incubated with HRP-conjugated IgG for 1 h at room temperature (25 °C). Finally, the membranes were scanned using a Tanon-5200 ECL chemiluminescent imaging system (Shanghai, China). Accordingly, the blot bands were quantitative analysis for statistical calculation.

### 2.9. Immunofluorescence

The cultural medium was discarded and washed with PBS twice. Cells were fixed in 4% paraformaldehyde solution for 30 min at room temperature (25 °C). The PBS was discarded and cells were mixed with 0.4% Triton X-100 solution for 10 min. The transmembrane solution was removed and combined with 5% BSA blocking buffer for 30 min at room temperature (25 °C). Cells were incubated with primary antibodies to Runx2 and IgG for 12 h, the latter serving as a negative control. We re-collected the primary antibodies and washed them with PBS 5 times. Then, the second immunofluorescence antibody was incubated for 2 h in the dark. Phalloidin staining was added into the cells for 10 min at room temperature (25 °C). Cells were washed with PBS 5 times and we added the Hoechst 33258 staining for 10 min at room temperature (25 °C). The images were recorded using a TCS SP8 STED high-resolution laser confocal microscope (Leica, Germany).

### 2.10. Statistical Analysis 

All experimental data were analyzed using a method of one-way analysis of variance (ANOVA) by SPSS 25.0 (IBM SPSS; Armonk, New York, NY, USA). All data are presented as mean ± standard deviation (SD). *p* < 0.05 and *p* < 0.01 indicated significant and extremely significant difference, respectively. All the experiments were performed in triplicate.

## 3. Results

### 3.1. The Effects of lα, 25-(OH)_2_D_3_ on Osteogenic Differentiation of BMSCs from Chickens 

**Figure 1:**
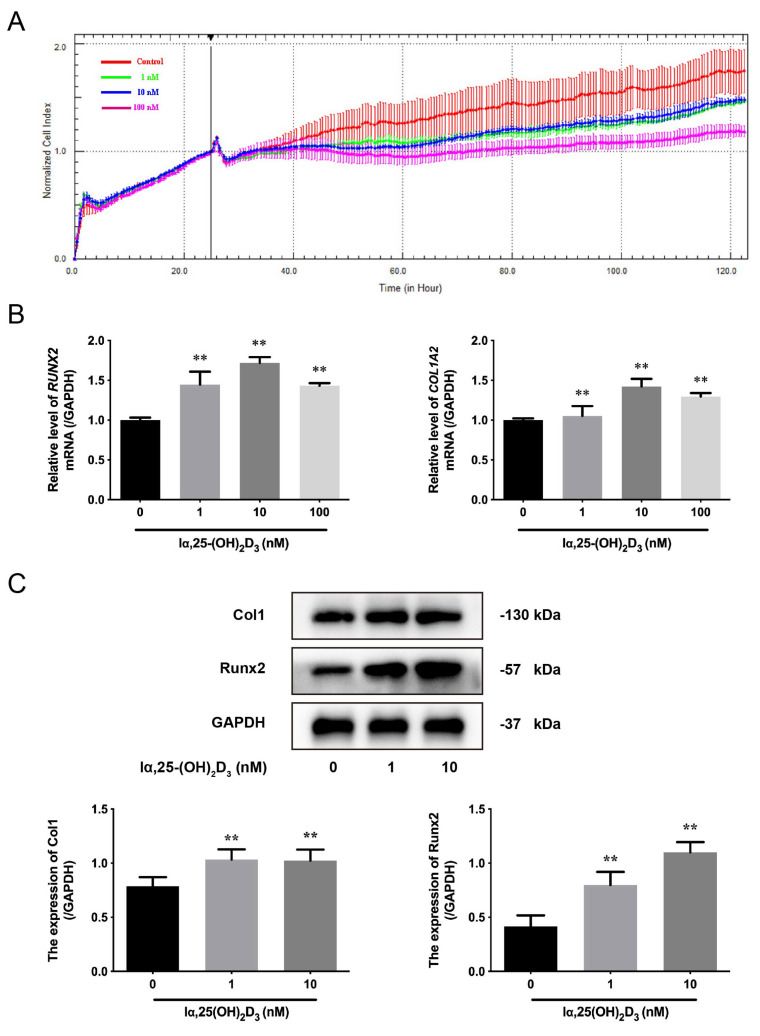
The effects of lα, 25-(OH)_2_D_3_ on osteogenic differentiation of BMSCs from chickens in vitro. (**A**) Cell index of BMSCs was monitored by the RTCA dynamic system after treatment with different concentrations (0, 1, 10, and 100 nmol/L) of lα, 25-(OH)_2_D_3_. (**B**) The mRNA levels of *RUNX2* and *COL1A2* in BMSCs were measured by qRT-PCR after treatment with different concentrations (0, 1, 10, and 100 nmol/L) of lα, 25-(OH)_2_D_3_. ** *p* < 0.01 vs. 0 nmol/L lα, 25-(OH)_2_D_3_. (**C**) The expression of Col1 and Runx2 in BMSCs were detected by Western blot after treatment with different concentrations (0, 1, and 10 nmol/L) of lα, 25-(OH)_2_D_3_. GAPDH was used as an internal reference protein. ** *p* < 0.01 vs. 0 nmol/L lα, 25-(OH)_2_D_3_.

**Figure 2:**
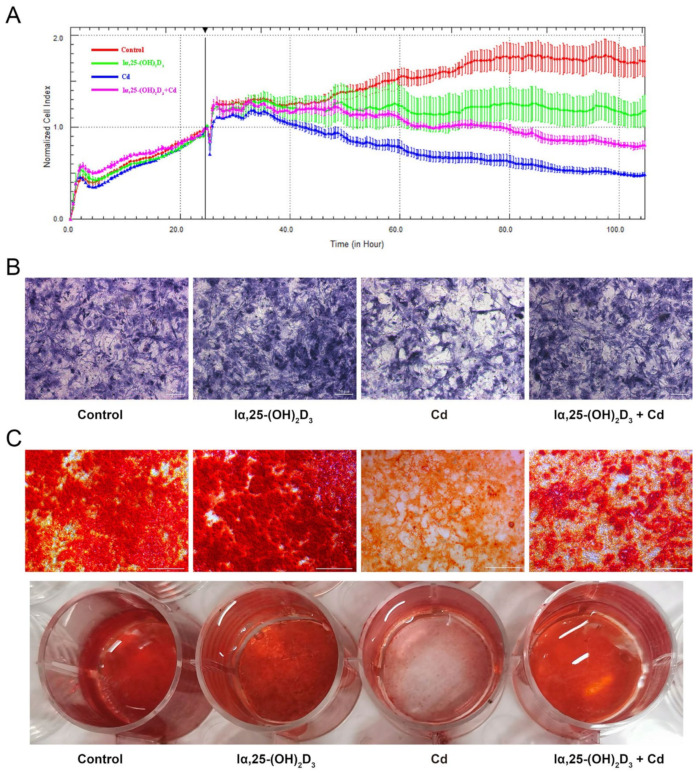
The effects of lα, 25-(OH)_2_D_3_ on Cd-inhibited osteogenic differentiation of BMSCs from chickens in vitro. (**A**) Cell index of BMSCs was monitored by the RTCA dynamic system after treatment with different groups. (**B**,**C**) The activity of ALP and the orange-red calcified nodules in BMSCs were observed using ALP staining and Alizarin red staining after treatment with different groups, respectively. (Control: only cells, lα, 25-(OH)_2_D_3_: 10 nmol/L; Cd: 5 μmol/L and lα, 25-(OH)_2_D_3_ + Cd: 10 nmol/L + 5 μmol/L, respectively).

It has been previously reported that lα, 25-(OH)_2_D_3_ has multiple regulatory effects, including cell proliferation and cell differentiation [20]. To detect the effects of lα, 25-(OH)_2_D_3_ on the proliferation of BMSCs from chicken embryos in vitro, the cell index was monitored using a RTCA dynamic system. The data showed that the BMSCs enters the logarithmic growth phase after treatment with lα, 25-(OH)_2_D_3_ for 24 h. Then, the cell index was observed with supplementation with different concentrations of lα, 25-(OH)_2_D_3_ (1, 10, and 100 nmol/L); the cell index was normalized to analyze the difference, and the cell index in the 100 nmol/L lα, 25-(OH)_2_D_3_ group was lower than the other groups. However, in terms of cell index, there was no difference between 1 and 10 nmol/L lα, 25-(OH)_2_D_3_ (Figure 1A). Next, the mRNA levels of *RUNX2* and *COL1A2* were measured by qRT-PCR after treatment with different concentrations of lα, 25-(OH)_2_D_3_; it was found that 10 nmol/L lα, 25-(OH)_2_D_3_, more than 10 nmol/L, significantly increased their levels compared with 0 nmol/L lα, 25-(OH)_2_D_3_ (Figure 2B). Correspondingly, the expression of Col1 and Runx2 were enhanced after treatment with 1 and 10 nmol/L lα, 25-(OH)_2_D_3_ compared with 0 nmol/L lα, 25-(OH)_2_D_3_ (Figure 2C). These data indicated that lα, 25-(OH)_2_D_3_ promoted the osteogenic differentiation of BMSCs from chickens.

### 3.2. The Effects of lα, 25-(OH)_2_D_3_ on Cd-Inhibited Osteogenic Differentiation of BMSCs from Chickens

To observe the proliferation of BMSCs treated by Cd, the cell proliferation, ALP staining and Alizarin red staining were utilized after different treatment in vitro. The results showed that 10 μmol/L Cd has a more obviously inhibitory effect than the control group, but 10 nmol/L lα, 25-(OH)_2_D_3_ displayed an alleviated action on the Cd-inhibited proliferation of BMSCs from chickens (Figure 2A). As shown in Figure 2B, the activity of ALP in the 10 nmol/L lα, 25-(OH)_2_D_3_ group was enhanced compared with the control group, while the 5 μmol/L Cd group decreased the purple black particles. However, the purple black particles in the 10 nmol/L lα, 25-(OH)_2_D_3_ co-treated with 5 μmol/L Cd group were higher than the 5 μmol/L Cd group. In addition, the color and stained area of orange-red calcified nodules in the 10 nmol/L lα, 25-(OH)_2_D_3_ group were enhanced using Alizarin red staining compared with the control group, while the 5 μmol/L Cd group decreased the color and stained area of orange-red calcified nodules. Compared with the 5 μmol/L Cd group, the color and stained area of orange-red calcified nodules in the the 10 nmol/L lα, 25-(OH)_2_D_3_ co-treated with the 5 μmol/L Cd group were restored (Figure 2C). These data demonstrated the alleviating effects of lα, 25-(OH)_2_D_3_ on Cd-inhibited osteogenic differentiation of BMSCs from chickens. 

### 3.3. The Distribution of Runx2 during lα, 25-(OH)_2_D_3_ on Cd-Induced Osteogenic Differentiation of BMSCs from Chickens 

**Figure 3:**
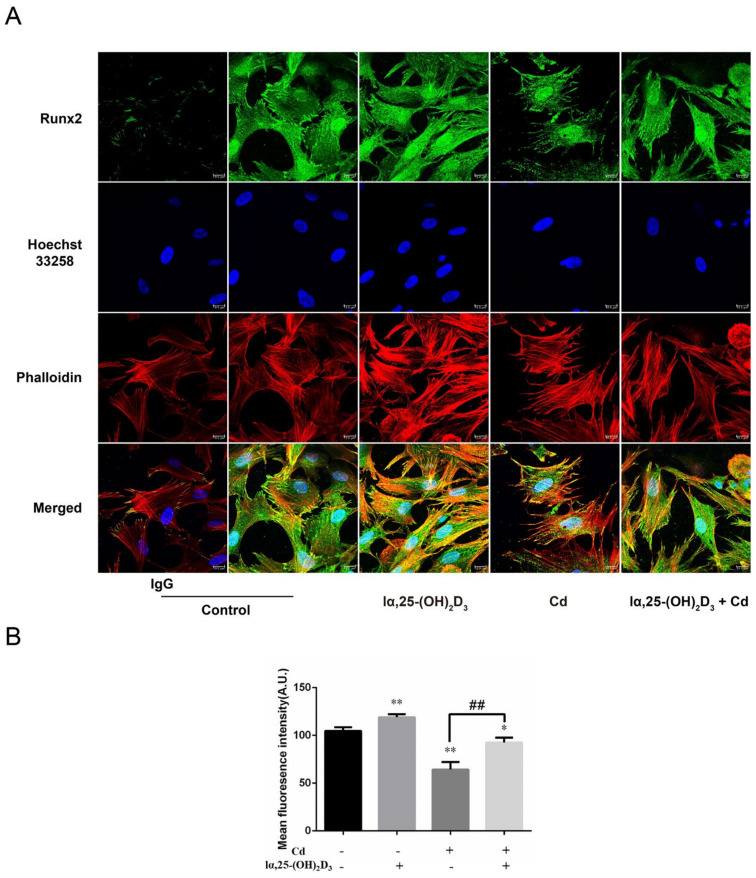
The distribution of Runx2 during lα, 25-(OH)_2_D_3_ on Cd-inhibited osteogenic differentiation of BMSCs from chickens in vitro. (**A**) Green fluorescence indicates the Runx2 distribution using a primary antibody, blue indicates the nucleus using the Hoechst 33258 dye, and red indicates the F-actin using a Phalloidin dye. (**B**) The mean fluorescence intensity of Runx2 was analyzed after treatment with different group. * *p* < 0.05, ** *p* < 0.01 vs. Control group; ## *p* < 0.01 vs. Cd group. (Control: only cells, lα, 25-(OH)_2_D_3_: 10 nmol/L; Cd: 5 μmol/L and lα, 25-(OH)_2_D_3_ + Cd: 10 nmol/L + 5 μmol/L, respectively).

As shown in Figure 3, the fluorescence intensity of Runx2 was enhanced after treatment with 10 nmol/L lα, 25-(OH)_2_D_3_ compared with the control group, and the IgG as a negative control. Furthermore, the 5 μmol/L Cd group significantly attenuated the Runx2 fluorescence intensity compared with the control group. The Runx2 fluorescence intensity was augmented in the 10 nmol/L lα, 25-(OH)_2_D_3_ co-treated with 5 μmol/L Cd group compared with the 5 μmol/L Cd group (Figure 3A,B). These data showed that lα, 25-(OH)_2_D_3_ can alleviate the inhibitory effect of Runx2 in Cd-induced osteogenic differentiation of BMSCs from chickens. 

### 3.4. The Effects of lα, 25-(OH)_2_D_3_ on Markers of Cd-Inhibited Osteogenic Differentiation of BMSCs from Chickens 

**Figure 4:**
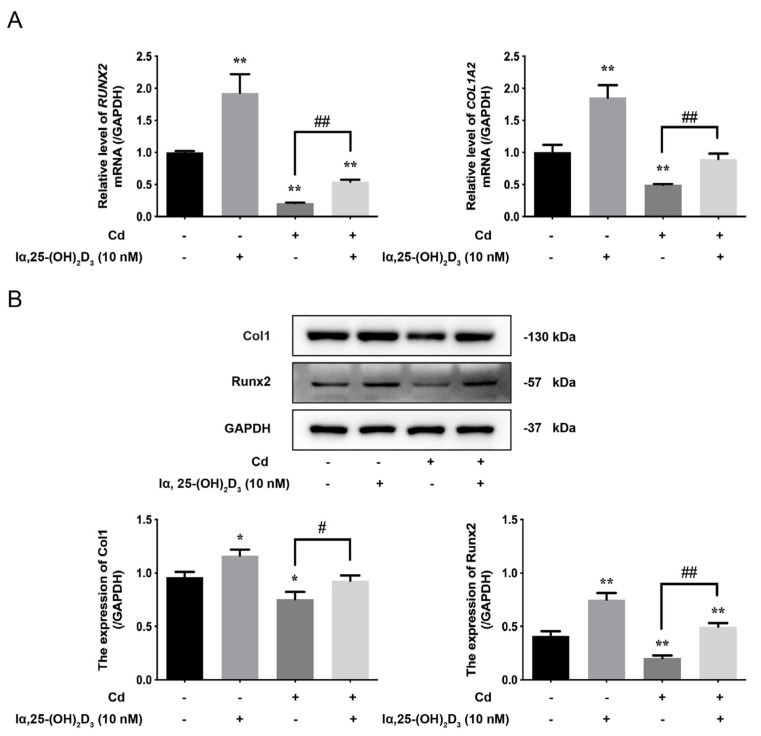
The effects of lα, 25-(OH)_2_D_3_ on markers of Cd-inhibited osteogenic differentiation of BMSCs from chickens in vitro. (**A**) The mRNA levels of *RUNX2* and *COL1A2* in BMSCs were measured by qRT-PCR after treatment with different groups. ** *p* < 0.01 vs. Control group; ## *p* < 0.01 vs. Cd group. (**B**) The expression of Col1 and Runx2 in BMSCs were detected by Western blot after treatment with different groups. GAPDH was used as an internal reference protein. * *p* < 0.05, ** *p* < 0.01 vs. Control group; # *p* < 0.05, ## *p* < 0.01 vs. Cd group. (Control: only cells, lα, 25-(OH)_2_D_3_: 10 nmol/L; Cd: 5 μmol/L and lα, 25-(OH)_2_D_3_ + Cd: 10 nmol/L + 5 μmol/L, respectively).

To further verify the osteogenic differentiation of BMSCs from chickens, the expression of osteoblastic markers was examined after different treatments in vitro. The results showed that the mRNA levels of *RUNX2* and *COL1A2* in the lα, 25-(OH)_2_D_3_ group were higher than the control group, but the 5 μmol/L Cd group decreased their levels. Compared with the 5 μmol/L Cd group, the levels of *RUNX2* and *COL1A2* mRNA in the 10 nmol/L lα, 25-(OH)_2_D_3_ co-treated with 5 μmol/L Cd group was augmented (Figure 4A). Correspondingly, the protein expression of Col1 and Runx2 showed the same trend with different treatments (Figure 4B). These results showed that lα, 25-(OH)_2_D_3_ can attenuate the inhibitory effects on the expression of markers in Cd-inhibited osteogenic differentiation of BMSCs from chickens.

### 3.5. The Effects of lα, 25-(OH)_2_D_3_ on Mitochondrial Membrane Potential and Apoptosis in Cd-Inhibited Osteogenic Differentiation of BMSCs from Chickens 

**Figure 5:**
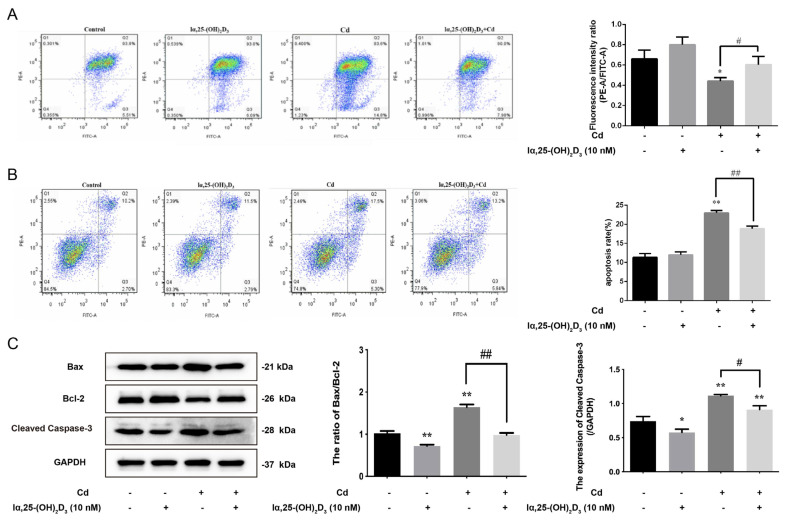
The effects of lα, 25-(OH)_2_D_3_ on mitochondrial membrane potential and apoptosis in Cd- inhibited osteogenic differentiation of BMSCs from chickens in vitro. (**A**,**B**) The mitochondrial membrane potential and apoptosis rate were measured by flow cytometry after treatment with different groups. ## *p* < 0.01, # *p* < 0.05 vs. Cd group. (**C**) the ratio of Bax/Bcl-2 and the expression of cleaved caspase-3 were detected by Western blot after treatment with different groups. GAPDH was used as an internal reference protein. * *p* < 0.05, ** *p* < 0.01 vs. Control group; ## *p* < 0.01 vs. Cd group. (Control: only cells, lα, 25-(OH)_2_D_3_: 10 nmol/L; Cd: 5 μmol/L and lα, 25-(OH)_2_D_3_ + Cd: 10 nmol/L + 5 μmol/L, respectively).

Next, the mitochondrial membrane potential of BMSCs was detected after different treatments in vitro; the results showed that there was no significant difference between lα, 25-(OH)_2_D_3_ group and the control group. However, 5 μmol/L Cd decreased the mitochondrial membrane potential of BMSCs. Furthermore, the mitochondrial membrane potential of BMSCs in the 10 nmol/L lα, 25-(OH)_2_D_3_ co-treated with 5 μmol/L Cd group was higher than that in the 5 μmol/L Cd group (Figure 5A). In addition, the apoptosis rate and apoptosis protein expression were measured after different treatments; the results showed that the apoptosis rate in the lα, 25-(OH)_2_D_3_ group had no change compared with the control group, but the apoptosis rate was increased in the 5 μmol/L Cd group. The apoptosis rate in the 10 nmol/L lα, 25-(OH)_2_D_3_ co-treated with 5 μmol/L Cd group was lower than the Cd group (Figure 5B). Correspondingly, the ratio of Bax/Bcl-2 was decreased in the lα, 25-(OH)_2_D_3_ group compared with the control group, but 5 μmol/L Cd increased the ratio of Bax/Bcl-2. The ratio of Bax/Bcl-2 and the expression of cleaved caspase-3 in the 10 nmol/L lα, 25-(OH)_2_D_3_ co-treated with 5 μmol/L Cd group were lower than the 5 μmol/L Cd group (Figure 5C). These data demonstrated that lα, 25-(OH)_2_D_3_ can alleviate the apoptosis in Cd-inhibited osteogenic differentiation of BMSCs from chickens. 

## 4. Discussion

Vitamin D_3_ is mainly produced by ultraviolet rays in the human skin (around 80–90%), and is also sourced from daily diet (around 2000–4000 IU on average) [37]. Vitamin D is a critical regulator for bone metabolism and Ca^2+^ balance by binding to its receptor VDR, and is involved in the differentiation and proliferation of cells, such as in the osteogenic differentiation of BMSCs [38,39]. Importantly, lα, 25-(OH)_2_D_3_, the principally active form of vitamin D, promoted the proliferation and migration of BMSCs in Sprague–Dawley (SD) rats [38]. lα, 25-(OH)_2_D_3_ has an up-regulated effect in hBMSCs or MC3T3-E1 osteoblastic cells through increasing the mRNA levels of *RUNX2*, *COL1A1* (a component of type I collagen alpha 1), *OCN*, and *ALP* [40,41]. Runx2 and Col1 are key osteogenic potential markers during osteoblast differentiation of gingiva-derived mesenchymal stem cells (GMSCs) [42]. Runx2 as a major biomarker of osteoblast differentiation, is a widely used transcription factor for osteoblasts [43,44]. Likewise, Col1 is also known as an early osteogenic marker, and its early expression represents an early stage of maturation [42,45]. Col1 has a triple helix comprising two alpha 1 chains and one alpha 2 chain [46]. However, the mutation of COL1A1 in induced pluripotent stem cells (iPSCs) from osteogenesis imperfecta (OI) may result in bone fragility and repeated fractures [40,47]. Correspondingly, the mutation of COL1A2 can cause Osteogenesis Imperfecta (OI), and is close to the central region of the helical chain; the osteoblast differentiation ability is affected [48,49,50]. In this study, the proliferation of BMSCs from chickens was suppressed after treatment with different concentrations (1, 10, and 100 nmol/L) of lα, 25-(OH)_2_D_3_. However, 10 nmol/L lα, 25-(OH)_2_D_3_ increased the mRNA levels of *RUNX2* and *COL1A2* and the protein expression of Runx2 and Col1 during osteogenic differentiation of BMSCs from chickens. These data are similar to those previously reported, while the species are different [40,41].

A previous study investigated that CdCl_2_ caused a reduction in cell activity in BMSCs from rats, nuclear breakage and chromatin condensation, and cytoplasm shrinkage [51]. A low concentration (0.1 or 0.2 µmol/L) of CdCl_2_ decreased the biomarker genes of bone marrow mesenchymal stem cells (BMMSCs) in SD rats, such as *ALP*, *RUNX2*, *OCN*, *osteopontin* (*OPN*), and *osterix* (*OSX*), as well as down-regulating the expression of ALP and Runx2, even ALP activity and the numbers of mineralization nodules, which were evaluated by ALP staining and Alizarin red staining, respectively [52]. Generally, the ALP staining was used to assess the ALP activity, which is also an early biomarker for osteogenic differentiation [52]. In addition, the mineralized nodules are usually observed at terminal differentiation, and are determined by Alizarin red staining as a late phase marker of osteogenic differentiation [53]. Consistent with ALP staining for decreasing the purple black particles, Cd reduced the orange-red mineralized nodules by Alizarin red staining. As Cd decreased the early and late marker of osteogenic differentiation, we further observed the distribution of Runx2 protein using immunofluorescence. It was found that Cd reduced the fluorescence intensity of Runx2 during the osteogenic differentiation of BMSCs from chickens. Correspondingly, Cd decreased the mRNA levels and the protein expression of osteogenic differentiation factors including Runx2 and Col1. Finally, the mitochondrial membrane potential of BMSCs from chickens was measured by flow cytometry; it was found that Cd reduced the mitochondrial membrane potential. As is well known, mitochondria dysfunction caused by a decrease in mitochondrial membrane potential is associated with cell apoptosis, including apoptosis rate, the abnormal expression of Bax/Bcl-2 ratio, and the activation of caspase-3 [54,55]. These induced mitochondria damages can lead to the apoptosis of BMSCs in osteogenic differentiation, which was consistent with studies on Cd toxicity in different species [32,56].

Next, we further explored the effects of lα, 25-(OH)_2_D_3_ on Cd-inhibited osteogenic differentiation of BMSCs from chickens. Several studies have shown that the repairing action of BMSCs in tissue injury is due to its localization in the bone marrow matrix, with the potential to differentiate and replace damaged cells and secrete cytokines to regulate cellular microenvironments [57,58]. For example, BMSCs repair the testicular injury of rats, and this may be highly related to mitochondrial apoptosis [57]. In addition, lα, 25-(OH)_2_D_3_ has a novel role in DNA repair in various tissues or in some disease processes, such as in breast cancer, and lα, 25-(OH)_2_D_3_ enhances the stabilization of the 53BP1 levels and the blockage of cathepsin L (a positive biomarker for triple-negative breast cancer) to treat or mitigate the tumorigenesis [59]. Our previous study demonstrated that lα, 25-(OH)_2_D_3_ palliated Cd-induced toxicity of chondrocytes in chicken embryo, are accompanied by the up-regulation of mitochondrial membrane potential and down-regulation of apoptosis rate and Bax/Bcl-2 ratio [33]. In this study, our data have shown that the lα, 25-(OH)_2_D_3_ increased the activity of ALP and orange-red calcified mineralized nodules, the distribution of Runx2, the levels of Runx2 and Col1 and mitochondrial membrane potential during Cd-inhibited osteogenic differentiation of chicken‘s BMSCs, while decreasing the apoptosis rate, Bax/Bcl-2 ratio, and activation of caspase-3. Therefore, these findings may provide new insight for repairing Cd-inhibited osteogenic differentiation of BMSCs from chickens.

**Figure 6:**
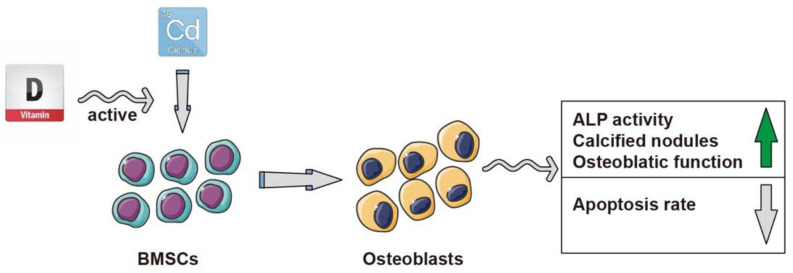
The schematic model of lα, 25-(OH)_2_D_3_, which facilitates the osteogenic differentiation and mineralization from chicken’s BMSCs, and alleviates Cd-induced toxicity of cells. Curved grey arrows represents mitigation effects, crooked arrows represent alleviating effects, and green straight arrow represents positive effects.

In conclusion, Cd has a toxicity role in BMSCs and/or osteogenic differentiation in chickens, and reduces osteogenic mineralization by promoting cell apoptosis in vitro. Moreover, lα, 25-(OH)_2_D_3_ facilitates the osteogenic differentiation and mineralization of BMSCs from chickens, and alleviates the Cd-induced toxicity of cells (Figure 6). However, the regulatory mechanisms by which 1α,25-(OH)_2_D_3_ mitigates Cd-stimulated toxicity need to be further investigated in detail.

## Figures and Tables

**Table 1:** The primer sequences of target genes for qRT-PCR.

Gene Name	Primer Sequence (5’–3’)	Gene Number	Length (bp)
*RUNX2*	Forward: AACCCAAACTTGCCCAACCA	NM_204128.2	114
	Reverse: AGTACGGCCTCCAAACGGA		
*COL1A2*	Forward: ATGGTCCTAGAGGTCTGCGT	NM_001079714.2	109
	Reverse: AGCCTCTCCAGATGGACCTT		
*GAPDH*	Forward: GGGTGGTGCTAAGCGTGTTA	NM_204305.2	180
	Reverse: ACCCTCCACAATGCCAAAGT		

## Data Availability

The raw data supporting the conclusions of this article will be made available by the authors. The origin western blot images presented in this study are available in Supplementary Materials.

## Supplementary Materials

The following supporting information can be downloaded at: https://www.mdpi.com/article/10.3390/ani13152544/s1, The origin western blot images of Col1, Runx2 and GAPDH in the Figure 1C; The origin western blot images of Col1, Runx2 and GAPDH in the Figure 4B; The origin western blot images of Bax, Bcl-2, Cleaved Caspase-3 and GAPDH in the Figure 5C.
