# Supplementary material for: Vitamin D Alleviates Cadmium-Induced Inhibition of Chicken Bone Marrow Stromal Cells’ Osteogenic Differentiation In Vitro
Source: Animals (Basel). 2023 Aug 7;13(15):2544. doi: 10.3390/ani13152544 (PMC10417335; doi:10.3390/ani13152544)
Supplement: Supplementary file 1 [file animals-13-02544-s001.zip › animals-2475796-supplementary.pdf]

Origin blots

Figure 1C

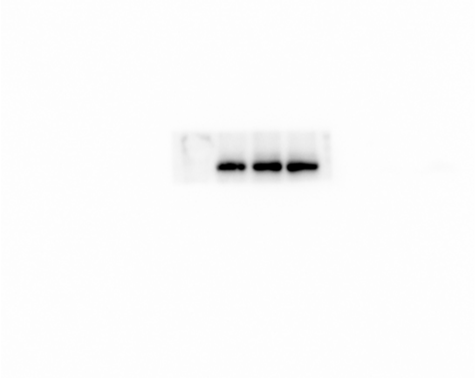

Col1 in Figure 1C

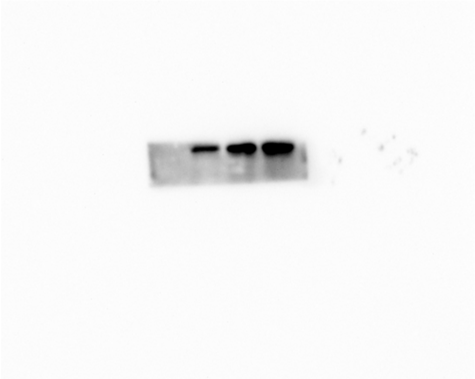

Runx2 in Figure 1C

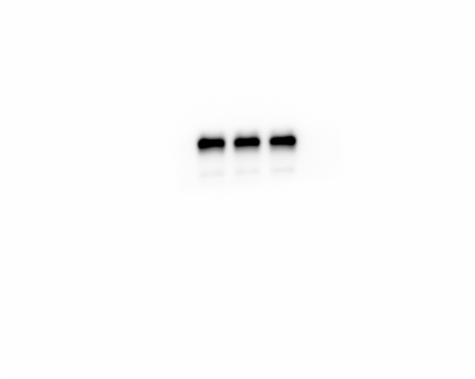

GAPDH in Figure 1C

Figure 4B

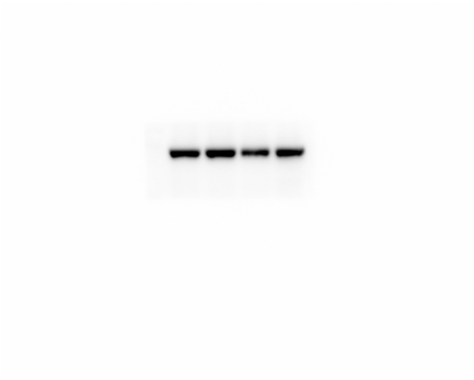

Col1 in Figure 4B

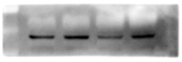

Runx2 in Figure 4B

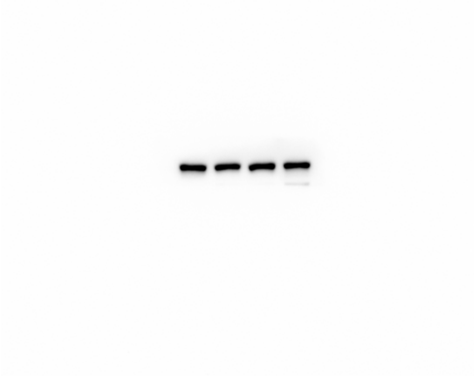

GAPDH in Figure 4B

Figure 5C

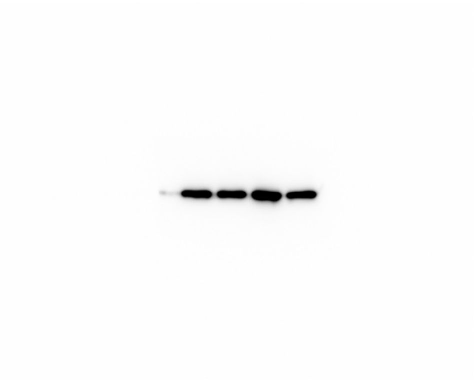

Bax in Figure 5C

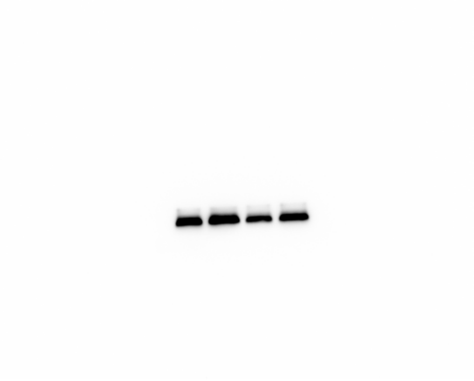

Bcl-2 in Figure 5C

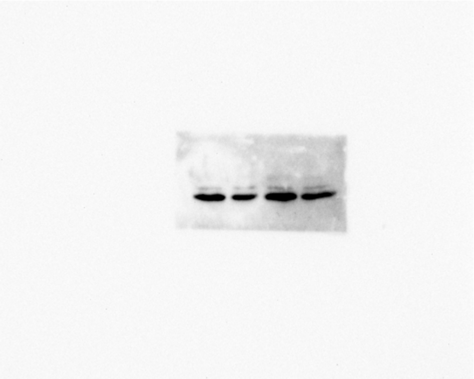

Cleaved Caspase-3 in Figure 5C

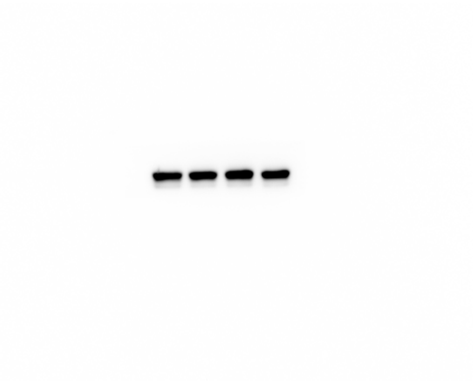

GAPDH in Figure 5C
